# Study on Pain Catastrophizing From 2010 to 2020: A Bibliometric Analysis via CiteSpace

**DOI:** 10.3389/fpsyg.2021.759347

**Published:** 2021-12-17

**Authors:** Huifang Luo, Zongliao Cai, Yanyi Huang, Jiating Song, Qing Ma, Xiangwei Yang, Yang Song

**Affiliations:** ^1^School of Nursing, Guangzhou University of Chinese Medicine, Guangzhou, China; ^2^Physical and Mental Medicine, Guangzhou Brain Hospital, Guangzhou, China; ^3^Second Clinical Medical College, Guangzhou University of Chinese Medicine, Guangzhou, China; ^4^Department of Rheumatology, The First Affiliated Hospital of Guangzhou University of Chinese Medicine, Guangzhou, China

**Keywords:** pain catastrophizing, bibliometric analysis, CiteSpace, research trends, Web of Science

## Abstract

**Purpose:** This study aimed to evaluate the global scientific output of research on pain catastrophizing and explore the hotspots and frontiers from 2010 to 2020 using bibliometric methods.

**Methods:** Publications regarding pain catastrophizing published from 2010 to 2020 were extracted from the Web of Science Core Collection. CiteSpace was used to analyze the number of publications, countries, institutions, journals, authors, cited references, and keywords using standard bibliometric indicators.

**Results:** A total of 1,576 publications on pain catastrophizing were retrieved from 2010 to December 31, 2020. The number and rate of the annual publications gradually increased totally. Pain (130) was the most productive journal. Meanwhile, Pain ranked first in the frequency (1,432) and centrality (0.31) of the cited journals. The most productive country and institution in this frequency field were the United States (642) and the University of Washington (73), respectively. Jensen MP (34) was the most prolific author, and Sullivan MJL (1,196) ranked first among the cited authors. In the ranking of frequency in the cited references, the first article was a critical review about pain catastrophizing published by Quartana (100). The keyword “Low back pain” had the highest frequency (556). “Total hip” was identified as a frontier research item for 2016–2020.

**Conclusion:** The findings of this bibliometric study provide the current status and trends in the clinical research of pain catastrophizing and may help researchers to identify hot topics and explore new research directions in this field.

## Introduction

Pain is listed as the fifth vital sign after body temperature, pulse, blood pressure, and respiration (Walid et al., 2008). With the deepening of the concept of the painless ward, the management of pain should be considered not only symptom management but also the psychological status of patients (Dansie and Turk, 2013). Psycho-social factors have been considered as important moderators and determinants of the pain experience in several chronic pain conditions (Innes, 2005; Quartana et al., 2009; Jensen et al., 2011; Giusti et al., 2020; Romeo et al., 2021). Several factors [e.g., kinesiophobia (Varallo et al., 2020, 2021a), pain acceptance (Esteve et al., 2007; Varallo et al., 2021b), pain vigilance (Roelofs et al., 2003)] have been identify as significant contributors to pain and disability, one of the most important is pain catastrophizing (Leung, 2012).

Pain catastrophizing is a set of negative irrational cognitions in the context of anticipated or actual pain (Sullivan et al., 2001). It is considered a belief system, a coping strategy, and an assessment process when an individual experiences and feels pain (Yap et al., 2008). It is characterized by three inadaptable dimensions—rumination (continuous negative thinking of pain), magnification (exaggerating the potential destructive power of pain), and helplessness (perception of their inability to cope with pain symptoms). It constitutes the most commonly considered psychosocial factor in predicting adjustment to chronic pain (De Baets et al., 2020). Besides, it is typically measured by the catastrophizing subscale of the 13-item Pain Catastrophizing Scale (PCS) developed by Sullivan MJL (Sullivan et al., 1995). The PCS has been translated into approximately 20 languages worldwide and is widely used in various patients with pain in Europe (Ikemoto et al., 2020; Majumder et al., 2020). In China, the most popular and widely used version of the PCS is the Hong Kong version (Yap et al., 2008).

Pain catastrophizing cannot be ignored. Penhoat has reported that patients with rheumatoid arthritis show high levels of pain catastrophizing even if they are treated with biological agents (Penhoat et al., 2014). Several studies have clearly demonstrated the incidence of pain catastrophizing. One previous study reported that the incidence of chronic pelvic pain catastrophizing was 53.1% (Sewell et al., 2018). Moreover, in a study of 11,214 patients with chronic pain, 39% patients reported severe pain catastrophizing (Brouwer et al., 2020). Evidence has suggested that pain catastrophizing has a consistently strong correlation with pain severity, disability, performance-based physical functioning and mood among people with chronic pain (Flor et al., 1993; Geisser et al., 1994; Thomtén et al., 2016; Salt et al., 2018; Varallo et al., 2021b). Individuals with pain catastrophizing tend to overreact to actual or potential pain, pay more attention to pain, and feel more incapable of coping with pain. In recent years, studies have confirmed that excessively high levels of pain catastrophizing make patients feel more pain, reduce pain tolerance, prolong the recovery period, increase the number of visits, and affect the treatment outcome (Jöud et al., 2017; Lazaridou et al., 2019; Martinez-Calderon et al., 2019).

Bibliometric is a quantitative statistical analysis tool that is used to analyze and observe research trends (Tran et al., 2018). It plays an important role in the theoretical and practical research of information science. With the method of bibliometrics, we can quickly clarity the characteristics of literature, analyze and grasp the development process and research hotspots of research fields. At present, bibliometric analysis is widely applied in various fields, including postpartum depression, acupuncture therapy on knee osteoarthritis, and stigma (Chen et al., 2020; Li et al., 2020; Bai et al., 2021). However, there is a lack of summary and evaluation on the literature characteristics, research direction, research depth, and hot spots of pain catastrophizing research. Therefore, it is essential to determine the current status of pain catastrophizing as a whole to provide the reference for future studies. In this study, we performed a bibliometric analysis of publications on pain catastrophizing by CiteSpace software from 2010 to 2020. Accordingly, we have provided an overview of the achievements and future research trends and hotpots in this research domain.

## Methods

### Data Acquisition

The bibliometric analysis relies on literature databases. At present, widely used databases include Scopus, Web of Science, Pubmed, Embased, Cochrane Library et al. Among them, the Web of Science database contains large-scale multidisciplinary, high-impact, international, and comprehensive academic journals. Meanwhile, evidence has shown that the Web of Science database provides a better knowledge map effect when CiteSpace is used for visual analysis (Falagas et al., 2008; Martín-Martín et al., 2018). Therefore, it is rational and effective to select the Web of Science database as our study data source. Specifically, the data were collected from the Web of Science Core Collection (WoSCC), including the SCI-EXPANDED, CCR-EXPANDED, and Index Chemicus. Due to the daily database updates, to avoid bias, we searched the literature retrieval from WoSCC on a single day, June 19, 2021.

The search strategy included the topic “pain catastrophizing” with the literature type limited to “ARTICLE” or “REVIEW.” Articles published from 2010 to 2020 in English were retrieved and we checked the relevance of the results. Finally, a total of 1,576 references were obtained. We saved the document data in the form of full records and cited references as plain text format. Some preparatory work is needed before data is imported into the CiteSpace. We created a new folder and created four folders in the folder named input, output, data, and project. Saved the file previously exported in WoSCC to input and name it “download _ ^**^.txt” format recognized by CiteSpace. The ^**^ represents the number. Then opened the CiteSpace to perform data format conversion and deduplication, visualization, and other operations. The results remained unchanged after data duplicate checking in the CiteSpace software.

### Analysis Tool

CiteSpace is a bibliometric analysis visualization software developed by Professor Chaomei Chen that uses the Java platform (Chen, 2004). It is an interactive analytic tool enabling visualization tasks in science mapping through a combination of bibliometrics, visual analytic methods, and data mining algorithms (Zhu et al., 2021). CiteSpace provides kinds of function selection for bibliometric studies, including collaboration network analysis, co-citation analysis, and co-occurrence analysis and can generate visual maps (Chen, 2017). By generating a series of visual knowledge maps, CiteSpace explores the research status, research hotspots, research frontiers, and evolution process of a scientific field, to reveal the research direction, research stage, and frontier characteristics of institutions and authors, and finally judge the development trend of this field.

The version of this software is constantly being updated. The version used in this research is 5.7 R1 (64-bit). The parameters of CiteSpace were as follows: time-slicing was performed from January 2010 to December 2020 (1 year per slice), all options in the term source were selected, one node type was selected at a time, selection criteria (top 50 objects), and pruning (pathfinder). Nodes and links were used to generate visualization knowledge maps. Each node in the map represented an element to be analyzed, such as a cited journal, country, or author. The size of the node represented the frequency of the citation, and nodes of different colors indicated different years. The connection lines between nodes were regarded as the co-occurrence or co-citation relationship; the lines’ thickness meant the strength of the relationship, and the color corresponded to the first co-occurrence or co-citation time of nodes. Colors from cool to warm represent early to recent. The centrality was also named betweenness centrality. Nodes with high centrality (> 0.1) were usually considered turning points or pivotal points in a field. When the CiteSpace default number of network nodes was greater than 350, the centrality calculation function would be closed. We need to manually click the “compute node centrality” function in the node menu.

## Results and Discussion

### Annual Publications

**FIGURE 1:**
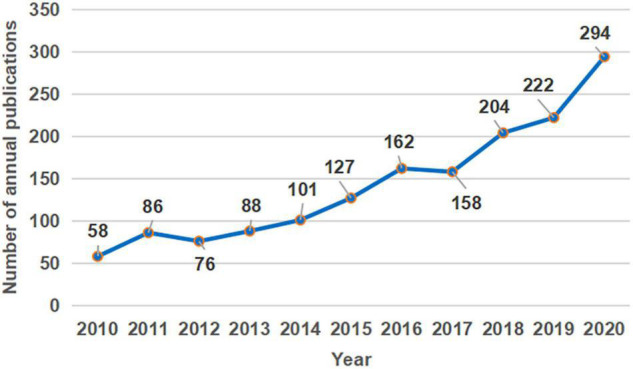
The number of pain catastrophizing research publications from 2010 to 2020.

A total of 1,576 publications were retrieved. Among these, 1,510 (95.81%) were research articles, 66 (4.19%) were review articles. The number of annual publications is shown in Figure 1. The number of publications increased from 58 in 2010 to 294 in 2020, but with some fluctuation. As shown in Figure 1, the trend of increase in publications can be divided into three stages: the initial stage (2010–2011), with a slow rate of publications; the smooth growth stage (2012–2016), with a slight continued upward trend; and the rapid growth stage (2017–2020), with accelerating output, and reached a peak in 2020.

### Analysis of Journals and Cited Journals

The number of journals that published 1,576 papers on pain catastrophizing research was 342. Many journals were pain-related specialized journals, other remaining journals were physical, psychological, and specialized diseases journals. The top five journals are listed by the number of publications in Table 1 and publishers of these journals were mostly located in the United States. The five journals all belong to the pain journal category. *Pain* ranked first in the frequency of the journals and the IF ranking. Only one journal’s impact factor exceeded 5, whereas the average impact factor of the remaining journals was approximately 3.38.

**TABLE 1:** The top five journals with the highest frequency of pain catastrophizing.

Ranking	Frequency	Journal*^a^*	IF 2020*^b^*	Country
1	130	*Pain*	5.483	Netherlands
2	104	*Clinical journal of pain*	2.893	United States
3	97	*Journal of pain*	4.621	United States
4	88	*Pain medicine*	2.513	United States
5	86	*European journal of pain*	3.492	England

*^a^Journal names according to Index of Medical Journal Abbreviations.*

*^b^IF in category according to Journal Citation Reports (2020).*

*IF, impact factor.*

Co-citation analysis, one of the most important indicators, has been widely applied in bibliometrics.

Co-cited journals were those cited together by other researchers. Through co-citation of journal analysis, we can obtain a distribution of key knowledge sources in a field. Table 2 presents the top five cited journals with the highest frequency and centrality of pain catastrophizing research. The most frequently cited journal was *Pain*, followed by *Clinical journal of pain*, *Psychological assessment*. In terms of centrality, the top five journals were *Physiotherapy research international*, *Biological psychiatry*, *Gastroenterology*, *American journal of industrial medicine*, *European spine journal*.

**TABLE 2:** The top five cited journals with the highest frequency and centrality of pain catastrophizing.

Ranking	Frequency	Cited journal^a^	IF 2020^b^	Country	Ran-king	Centrality	Cited journal^a^	IF 2020^b^	Country
1	1,432	*Pain*	5.483	Netherlands	1	0.31	*Physiotherapy research international*	/	United States
2	1,119	*Clinical journal of pain*	2.893	United States	2	0.27	*Biological psychiatry*	12.095	United States
3	1,102	*Psychological assessment*	2.825	United States	3	0.21	*Gastroenterology*	17.373	United States
4	1,069	*Journal of pain*	4.621	United States	4	0.19	*American journal of industrial medicine*	1.739	United States
5	890	*European journal of pain*	3.492	England	5	0.18	*European spine journal*	2.458	United States

*^a^Journal names according to Index of Medical Journal Abbreviations.*

*^b^IF in category according to Journal Citation Reports (2020).*

*IF, impact factor.*

### Distribution of Countries and Institutions

**FIGURE 2:**
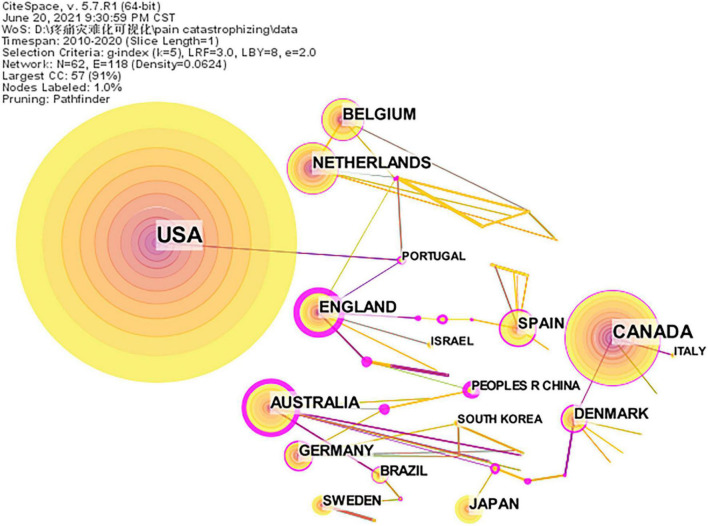
A country cooperation map related to pain catastrophizing from 2010 to 2020. The nodes in the map represent countries. The lines between the nodes represent cooperation relations.

A distribution map of countries was generated; the merged network comprised 62 nodes and 118 links (Figure 2). The nodes and links between them reveal the countries and cooperative relationships, respectively. The large the node, the more publications. In addition, the wider the line, the stronger the relationships. In total, 1,576 references were published by research groups in 62 countries; the top five countries were the United States, Canada, Netherlands, Australia, and Belgium (Table 3). In total, 642 articles were published in the United States, therefore, it was in the top position in terms of the research on pain catastrophizing. Pain is a significant public health concern in the United States. The United States, Canada, Netherlands, Belgium were the most prolific countries in Northern America and Europe, while Australia was the most productive country in Oceania. The top five countries in terms of centrality were England (1.05), Sri Lanka (0.98), Scotland (0.91), Australia (0.89), and China (0.86). The centrality of these five countries is greater than 0.1, indicating that these five countries have a certain influence in the study of pain catastrophizing.

**TABLE 3:** The top five countries related to pain catastrophizing.

Ranking	Frequency	Country	Ranking	Centrality	Country
1	642	United States	1	1.05	England
2	220	Canada	2	0.98	Sri Lanka
3	123	Netherlands	3	0.91	Scotland
4	119	Australia	4	0.89	Australia
5	109	Belgium	5	0.86	Peoples R China

**FIGURE 3:**
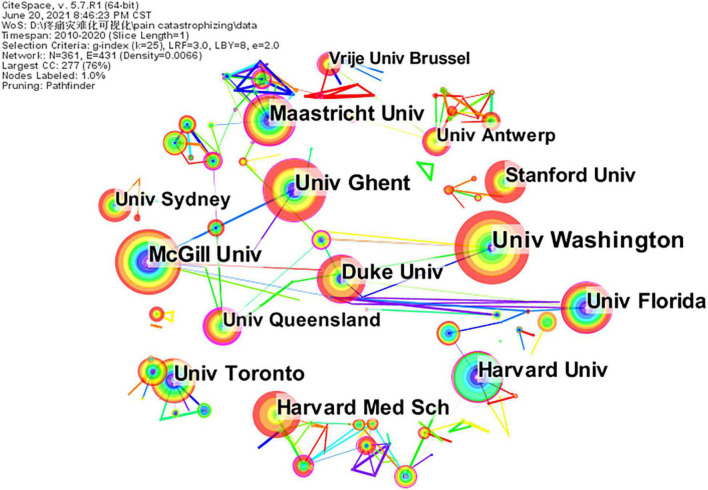
An institution cooperation map related to pain catastrophizing research from 2010 to 2020.

A distribution map of institutions was generated; the merged network comprised 361 nodes and 431 links (Figure 3). The 1,576 references were published in 361 institutions; the top five institutions were the University of Washington, McGill University, Ghent University, University of Florida, and Maastricht University (Table 4). The top five institutions in terms of centrality were The University of Queensland (0.41), Queen’s University (0.38), Auckland University of Technology (0.37), Brigham and Womens Hospital (0.33), and Aarhus University Hospital (0.29). Generally speaking, institutions were mainly concentrated in universities and a few in hospitals.

**TABLE 4:** The top five institutions related to pain catastrophizing.

Ranking	Frequency	Institution	Ranking	Centrality	Institution
1	73	University of Washington	1	0.41	The University of Queensland
2	64	McGill University	2	0.38	Queen’s University
3	62	Ghent University	3	0.37	Auckland University of Technology
4	52	University of Florida	4	0.33	Brigham and Womens Hospital
5	50	Maastricht University	5	0.29	Aarhus University Hospital

### Analysis of Authors and Cited Authors

**FIGURE 4:**
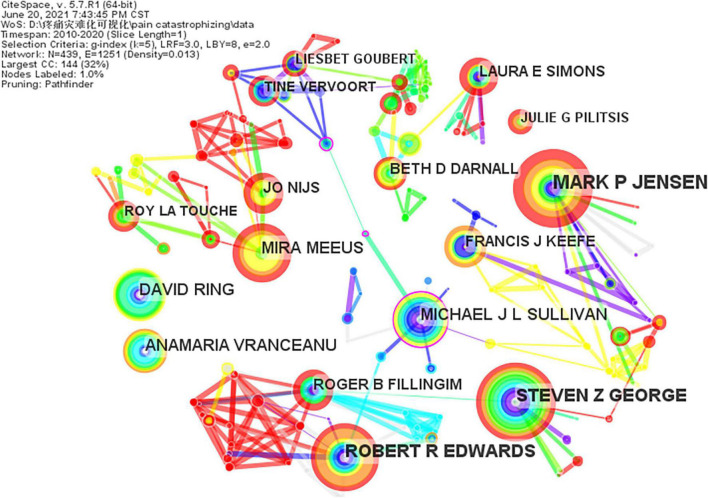
A co-author map related to pain catastrophizing research from 2010 to 2020.

Concerning the number of publications, Jensen MP from the University of Washington was the most prolific author. One of his articles identified a biological factor that may be associated with greater vulnerability to pain-related catastrophizing, which may contribute a new possibility for treating catastrophizing (Jensen et al., 2015). George SZ, Edwards RR, Meeus M, and Ring D were also active in the field of pain catastrophizing research (Table 5). As shown in Figure 4, the map of the authors comprised 439 nodes and 1,251 links. The size of the nodes in the cooperation graph represents the number of articles published by the authors, and the thickness of the lines between them reflects the strength of their cooperation relationship.

**TABLE 5:** The top five authors and cited authors related to pain catastrophizing.

Ranking	Frequency	Author	Ranking	Centrality	Author	Ranking	Frequency	Cited author	Ranking	Centrality	Cited author
1	34	Jenson MP	1	0.13	Sullivan MJL	1	1,196	Sullivan MJL	1	0.3	Staud R
2	33	George SZ	2	0.11	Trost Z	2	335	Edwards RR	2	0.29	Katz J
3	30	Edwards RR	3	0.11	Milioto M	3	334	OSMAN A	3	0.23	Walker LS
4	24	Meeus M	4	0.07	Edwards RR	4	330	Vlaeyen JWS	4	0.23	Arendt-NielsenlI L
5	24	Ring D	5	0.07	Meeus M	5	307	Crombez G	5	0.23	Zale EL

CiteSpace was used to generate a co-author map comprised of 653 nodes and 829 links. Sullivan MJL ranked the highest about citation counts (1,196), followed by Edwards RR (335), Osman A (334), Vlaeyen JWS (330), and Crombez G (307) (Table 5). From the cluster summary, the authors devoted their mind to scale development, mechanism exploration, related outcomes report, intervention research of pain catastrophizing. Sullivan MJL, a specialist in psychology from McGill University, focused on the development of the PCS and the outcome of pain catastrophizing, such as poor response to disease and poor quality of life. The top five cited authors in centrality were Staud R, Katz J, Walker LS, Arendt-NielsenlI L, and Zale EL.

### Analysis of Cited References

The top five cited references about frequency and centrality are shown in Tables 6, 7. According to the ranking of frequency and centrality in cited references, most were review papers, a few were original research papers. The first ranked citation in terms of frequency was the article published in *Expert Review of Neurotherapeutics* titled, “Pain catastrophizing: a critical review” (Quartana et al., 2009). In this review, Quartana PJ provided a detailed explanation on the conceptualization of pain catastrophizing, focused the discussion on a number of theoretical mechanisms of action and offered evidence to show that pain catastrophizing represents an important process factor in pain treatment.

**TABLE 6:** The top five cited references for the highest frequency of pain catastrophizing.

Ranking	Frequency	Cited reference	Representative author (publication year)
1	100	Pain catastrophizing: a critical review	Quartana, 2009
2	71	The Fear-avoidance model of musculoskeletal pain: current state of scientific evidence	Leeuw et al., 2007
3	58	Pain, catastrophizing, and depression in the rheumatic diseases	Edwards et al., 2011
4	49	Central sensitization: implications for the diagnosis and treatment of pain	Woolf, 2011
5	48	Pain catastrophizing in children with chronic pain and their parents: Proposed clinical reference points and reexamination of the PCS measure	Pielech et al., 2014

**TABLE 7:** The top five cited references for the highest centrality of pain catastrophizing.

Ranking	Centrality	Cited reference	Representative author (publication year)
1	0.23	Understanding the co-occurrence of anxiety disorders and chronic pain: state-of-the-art	Asmundson and Katz, 2009
2	0.22	Parental emotional responses to their child’s pain: the role of dispositional empathy and catastrophizing about their child’s pain	Goubert et al., 2008
3	0.2	The relationship of demographic and psychosocial variables to pain-related outcomes in a rural chronic pain population	Day and Thorn, 2010
4	0.2	Pain, catastrophizing, and depression in the rheumatic diseases	Edwards et al., 2011
5	0.19	Parental catastrophizing about child’s pain and its relationship with activity restriction: the mediating role of parental distress	Caes et al., 2011

**FIGURE 5:**
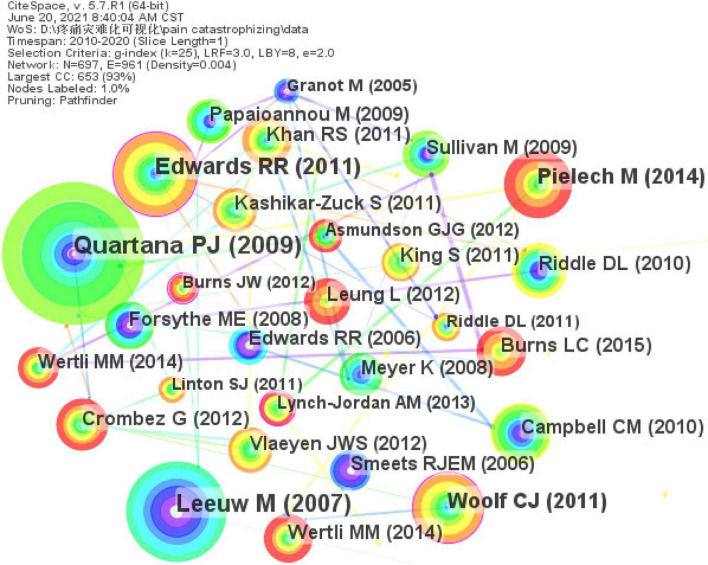
Reference co-citation map related to pain catastrophizing research from 2010 to 2020.

About the centrality of the cited references, the study that was ranked first was conducted by Asmundson, a Ph.D. in psychology from Canada. In this review, the author described the current state-of-the-art regarding the co-occurrence of anxiety disorders and chronic pain and pointed out that pain-related catastrophizing may maintain or exacerbate clinically significant symptoms of anxiety (Asmundson and Katz, 2009). The network map of the cited references comprised 697 nodes and 961 links (Figure 5). Over the past decade, a comprehensive analysis referring to multiple studies mainly focused on the following: the concept of pain catastrophizing analysis; its related mechanism exploration and outcomes report; the development, reliability, and validity test and localization of the PCS to adapt it to the cultures of various countries in the world; and the tentative exploration of intervention measures to reduce pain catastrophizing.

### Analysis of Keywords

**FIGURE 6:**
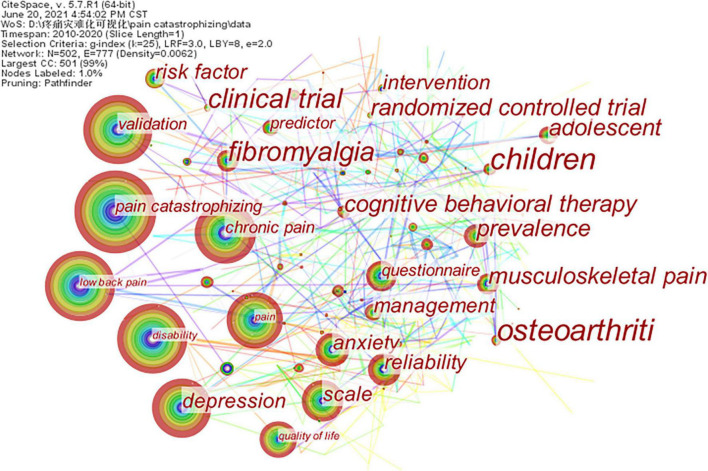
A keyword co-occurrence map of pain catastrophizing research from 2010 to 2020.

It was believed that research frontiers could be identified by analyzing the frequency and centrality of keywords. The map of keyword co-occurrence was generated; it consisted of 502 nodes and 777 links (Figure 6). We found that “low back pain” was the most popular keyword after removing the “pain catastrophizing” word. Low back pain is one of the most frequent problems worldwide, with an estimated global prevalence of 40–85%, and is the leading cause of functional decline and disability (Tagliaferri et al., 2020). A survey by Coyne also demonstrated that among 809 participants, the most common pain condition was lower back pain, with an incidence rate of 76.6% (Coyne et al., 2021). It is commonly believed that most people with low back pain can recover in a few weeks. However, reoccurrences are common and considerable fractions may go on to develop chronic low back pain. The occurrence of low back pain episodes mainly accounts for biomechanical factors, but psychosocial factors seem to take an important place in chronicity (Ibrahim et al., 2021). One important psychological factor linked with chronic low back pain is catastrophizing. Pain catastrophizing can predict the development of chronic back pain within one year after painless baseline and chronification of acute back pain (Taub et al., 2017). According to the frequency and centrality, the other popular keywords were “validation,” “disability,” “chronic pain,” “depression,” “quality of life,” “anxiety,” “attention” (Table 8).

**TABLE 8:** The top 10 keywords related to pain catastrophizing.

Ranking	Frequency	Keyword	Ranking	Centrality	Keyword
1	414	Pain catastrophizing	1	0.16	Attention
2	353	Low back pain	2	0.16	Disability index
3	353	Validation	3	0.16	Cognitive behavioral treatment
4	347	Disability	4	0.16	Endurance
5	309	Chronic pain	5	0.13	Mindfulness
6	304	Depression	6	0.13	Depressive symptom
7	298	Pain	7	0.12	Postoperative pain
8	221	Scale	8	0.12	Self-efficacy
9	200	Quality of life	9	0.12	Coping strategy
10	179	Anxiety	10	0.12	Fear avoidance belief

**FIGURE 7:**
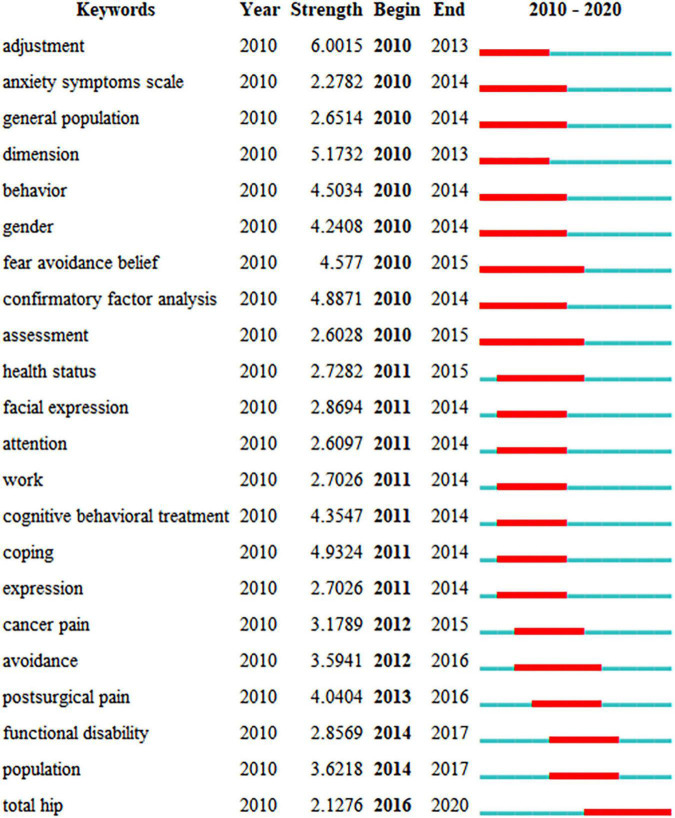
Top 22 keywords with the strongest citation bursts.

“Burst words” mean that words are cited frequently over some time. We can predict the research frontier according to the distribution of keywords with the strongest citation burst. The top 22 keywords with the strongest citation burst from 2010 to 2020 are shown in Figure 7. The red bars demonstrated that the keyword was cited frequently, the green bars showed that the keyword was cited infrequently. Functional disability, population, and total hip would be potentially cited frequently over the coming years, which represent the emerging trends.

1. Functional disability: Due to expectations of pain, patients who do not participate in activities may experience a considerable rise in functional disability over time (Millere et al., 2020). Pain catastrophizing contributed significant variance to the prediction of functional disability. A reduction in pain catastrophizing will lead to a reduction in pain and disability (Sullivan et al., 2005).
2. Population: Population is an epidemiological study of specific populations. It includes ordinary normal people and patients with certain diseases. A follow-up study of the general population for more than 4 years was carried out to observe the occurrence and development of chronic pain, and it was concluded that pain catastrophizing was an important predictor of moderate to severe chronic pain in the future (Landmark et al., 2018). The PCS is continuously developing and improving, and some researchers have conducted cross-cultural adaptation to the local general population and patients (Süren et al., 2014; Majumder et al., 2020).
3. Total hip: The hip is considered of the main load-bearing joints of the body. Total hip arthroplasty (THA) has become a common treatment for patients with severe hip functional impairment, and it is considered one of the most successful and cost-effective operations in modern medicine (Chen et al., 2021). It is estimated that by 2030, the incidence of THA will increase by approximately 174% (Kurtz et al., 2007). Nevertheless, postoperative pain after THA often persists for many years (Hayashi et al., 2018). Pain is experienced vary from person to person and can be exaggerated potentially limiting outcomes following hip and knee arthroplasty in some circumstances. Patients who catastrophize have been shown to have higher pain (Wood et al., 2021).

## Conclusion

It is clear from the bibliometric analysis of pain catastrophizing over the past 10 years that in general, the number of related publications is increasing. In this study, *Pain* ranked first in the frequency of the journals and cited journals, which revealed that the research achievements of this journal on pain catastrophizing have been affirmed by many researchers. A majority of journals are in the field of pain, the rest are in the field of psychology, pain-related diseases. The United States, Canada, and England, with a high publication rate and centrality, were the main research powers in this field. As is shown in Figure 2, it showed that developed countries, such as the United States and the United Kingdom, were the main countries researching on pain catastrophizing and have formed the power of small group cooperation, but there was a lack of cooperation between countries, as well as cooperation between the United States—the largest number of publications and other countries was less and lack of communication and sharing. The same was true of the organization, just formed their research team. The University of Washington was a major research institution. Most institutions were mainly concentrated in universities and a few in hospitals. Therefore, multi-country and multi-institution exchanges and cooperation should be strengthened to exchange and share the research results of pain catastrophizing in different regions and disciplines, and promote the continuous progress of research. The most prolific author was Jensen MP from the United States, while Sullivan MJL was the top cited author, primarily due to his first definition of pain catastrophizing and the development of a widespread PCS. According to the cited references, most were reviews on the conceptualization of pain catastrophizing and discussion on the related mechanism and model.

We analyzed the frequency and centrality of the keywords. The results showed that the research hotspots can be summarized as follows:

(1) Patients with low back pain and chronic pain were the main groups of pain catastrophizing research. In addition, it also involved patients in the field of rheumatism, cancer patients, postoperative patients, children, and so on. The research population was gradually diversified and enriched.
(2) The development of PCS evaluation tools covered framework design, dimension determination, reliability, and validity test, and focused on cross-cultural adaptation research under different populations and cultural backgrounds. This corresponded to the term “population” at the forefront of research.
(3) Study on the relationship between pain catastrophizing and health-related indicators, including the relationship between pain catastrophizing and depression, anxiety, quality of life, disability, and well-being.
(4) Research on the strategy of pain catastrophizing improvement. Cognitive-behavioral treatment has been examined as an effective way to decrease high levels of pain catastrophizing (Lazaridou et al., 2017; Scarone et al., 2020). A randomized controlled trial was a good method to test its effectiveness. But most studies considered pain catastrophizing as the secondary outcome indicator (Birch et al., 2020; Scarone et al., 2020). Therefore, future research should focus on matching interventions with specific dimensions of pain catastrophizing to improve the intervention effect. Of note, some studies suggested that cognitive behavioral therapy didn’t affect the level of pain catastrophizing (Birch et al., 2020). This was a controversial point. The future is worth further study.

In addition, a burst keyword can reflect cutting-edge research topics. Research history of pain catastrophizing can be extracted from the evolution of keywords used in these papers. For example, “adjustment,” “dimension,” and “confirmatory factor analysis” were the strongest citation bursts in the earlier to have an impact, which revealed that early research frontiers focused on emotional psychological adjustment and scale reliability and validity test of pain catastrophizing. “Total hip” was widely carried out in 2016 and has since been used. It is anticipated that the research of pain catastrophizing in the field of orthopedics is a continuing mainstream trend.

In conclusion, this study provides valuable information on potential collaborators and institutions, thereby providing an insight into the developing trend of pain catastrophizing research, which may guide new directions for further study.

## Author Contributions

HL and ZC designed the study, retrieved the data, performed the statistical analysis, and wrote the first draft. YH made the further modifications. JS and QM processed the figures and tables. XY and YS supervised the whole process and provided modification advice. All authors contributed to the article and approved the submitted version.

## Conflict of Interest

The authors declare that the research was conducted in the absence of any commercial or financial relationships that could be construed as a potential conflict of interest.

## Publisher’s Note

All claims expressed in this article are solely those of the authors and do not necessarily represent those of their affiliated organizations, or those of the publisher, the editors and the reviewers. Any product that may be evaluated in this article, or claim that may be made by its manufacturer, is not guaranteed or endorsed by the publisher.

## Abbreviations

WoSCC: Web of Science Core Collection
PCS: Pain Catastrophizing Scale.

## References

[B1] Asmundson G. J. Katz J. (2009). Understanding the co-occurrence of anxiety disorders and chronic pain: state-of-the-art. *Depress. Anxiety* 26 888–901. 10.1002/da.20600 19691031

[B2] Bai X. Song Z. Zhou Y. Wang X. Wang Y. Zhang D. (2021). Bibliometrics and Visual Analysis of the Research Status and Trends of Postpartum Depression From 2000 to 2020. *Front. Psychol.* 12:665181. 10.3389/fpsyg.2021.665181 34108920 PMC8180864

[B3] Birch S. Stilling M. Mechlenburg I. Hansen T. B. (2020). No effect of cognitive behavioral patient education for patients with pain catastrophizing before total knee arthroplasty: a randomized controlled trial. *Acta Orthop.* 91 98–103. 10.1080/17453674.2019.1694312 31762342 PMC7006640

[B4] Brouwer B. Waardenburg S. Jacobs C. Overdijk M. Leue C. Köke A. (2020). Biopsychosocial baseline values of 15 000 patients suffering from chronic pain: Dutch DataPain study. *Reg. Anesth. Pain Med.* 45 774–782. 10.1136/rapm-2020-101476 32784227

[B5] Caes L. Vervoort T. Eccleston C. Vandenhende M. Goubert L. (2011). Parental catastrophizing about child’s pain and its relationship with activity restriction: the mediating role of parental distress. *Pain* 152 212–222. 10.1016/j.pain.2010.10.037 21126822

[B6] Chen C. (2004). Searching for intellectual turning points: progressive knowledge domain visualization. *Proc. Natl. Acad. Sci. U S A.* 101(Suppl. 1), 5303–5310. 10.1073/pnas.0307513100 14724295 PMC387312

[B7] Chen C. (2017). Science mapping: a systematic review of the literature. *J. Data Informat. Sci.* 2 1–40. 10.1515/jdis-2017-0006

[B8] Chen S. Lu Q. Bai J. Deng C. Wang Y. Zhao Y. (2020). Global publications on stigma between 1998-2018: A bibliometric analysis. *J. Affect. Disord.* 274 363–371. 10.1016/j.jad.2020.05.006 32469828

[B9] Chen Z. Li B. Chen K. Feng J. Wang Y. Liu Z. (2021). Malalignment and distal contact of short tapered stems could be associated with postoperative thigh pain in primary total hip arthroplasty. *J. Orthop. Surg. Res.* 16:67. 10.1186/s13018-021-02215-w 33468189 PMC7816452

[B10] Coyne K. S. Barsdorf A. I. Currie B. M. Poon J. L. Mazière J. Y. Pierson R. F. (2021). Insight into chronic pain in the United States: descriptive results from the Prescription Opioid Misuse and Abuse Questionnaire (POMAQ) validation study. *Curr. Med. Res. Opin.* 37 483–492. 10.1080/03007995.2020.1865889 33331191

[B11] Dansie E. J. Turk D. C. (2013). Assessment of patients with chronic pain. *Br. J. Anaesthes.* 111 19–25. 10.1093/bja/aet124 23794641 PMC3841375

[B12] Day M. A. Thorn B. E. (2010). The relationship of demographic and psychosocial variables to pain-related outcomes in a rural chronic pain population. *Pain* 151 467–474. 10.1016/j.pain.2010.08.015 20817401 PMC2962925

[B13] De Baets L. Matheve T. Timmermans A. (2020). The association between fear of movement, pain catastrophizing, pain anxiety, and protective motor behavior in persons with peripheral joint conditions of a musculoskeletal origin: a systematic review. *Am. J. Phys. Med. Rehabil.* 99 941–949. 10.1097/PHM.0000000000001455 32349043

[B14] Edwards R. R. Cahalan C. Mensing G. Smith M. Haythornthwaite J. A. (2011). Pain, catastrophizing, and depression in the rheumatic diseases. *Nat. Rev. Rheumatol.* 7 216–224. 10.1038/nrrheum.2011.2 21283147

[B15] Esteve R. Ramírez-Maestre C. López-Marínez A. E. (2007). Adjustment to chronic pain: the role of pain acceptance, coping strategies, and pain-related cognitions. *Ann. Behav. Med.* 33 179–188. 10.1007/BF02879899 17447870

[B16] Falagas M. E. Pitsouni E. I. Malietzis G. A. Pappas G. (2008). Comparison of PubMed, Scopus, Web of Science, and Google Scholar: strengths and weaknesses. *Faseb J.* 22 338–342. 10.1096/fj.07-9492LSF 17884971

[B17] Flor H. Behle D. J. Birbaumer N. (1993). Assessment of pain-related cognitions in chronic pain patients. *Behav. Res. Ther.* 31 63–73. 10.1016/0005-7967(93)90044-u 8417730

[B18] Geisser M. E. Robinson M. E. Keefe F. J. Weiner M. L. (1994). Catastrophizing, depression and the sensory, affective and evaluative aspects of chronic pain. *Pain* 59 79–83. 10.1016/0304-3959(94)90050-7 7854806

[B19] Giusti E. M. Manna C. Varallo G. Cattivelli R. Manzoni G. M. Gabrielli S. (2020). The Predictive Role of Executive Functions and Psychological Factors on Chronic Pain after Orthopaedic Surgery: A Longitudinal Cohort Study. *Brain Sci.* 10:685. 10.3390/brainsci10100685 32998214 PMC7601771

[B20] Goubert L. Vervoort T. Sullivan M. J. Verhoeven K. Crombez G. (2008). Parental emotional responses to their child’s pain: the role of dispositional empathy and catastrophizing about their child’s pain. *J. Pain* 9 272–279. 10.1016/j.jpain.2007.11.006 18206424

[B21] Hayashi K. Kako M. Suzuki K. Takagi Y. Terai C. Yasuda S. (2018). Impact of variation in physical activity after total joint replacement. *J. Pain Res.* 11 2399–2406. 10.2147/JPR.S178853 30425553 PMC6200437

[B22] Ibrahim A. A. Akindele M. O. Kaka B. Mukhtar N. B. (2021). Development of the Hausa version of the Pain Catastrophizing Scale: translation, cross-cultural adaptation and psychometric evaluation in mixed urban and rural patients with chronic low back pain. *Health Qual. Life Out.* 19:44. 10.1186/s12955-020-01644-1 33546701 PMC7863472

[B23] Ikemoto T. Hayashi K. Shiro Y. Arai Y. C. Marcuzzi A. Costa D. (2020). A systematic review of cross-cultural validation of the pain catastrophizing scale. *Eur. J. Pain* 24 1228–1241. 10.1002/ejp.1587 32416018

[B24] Innes S. I. (2005). Psychosocial factors and their role in chronic pain: A brief review of development and current status. *Chiropract. Osteopathy* 13:6. 10.1186/1746-1340-13-6 15967055 PMC1151654

[B25] Jensen M. P. Gianas A. Sherlin L. H. Howe J. D. (2015). Pain Catastrophizing and EEG-α Asymmetry. *Clin. J. Pain* 31 852–858. 10.1097/AJP.0000000000000182 25411858 PMC4437922

[B26] Jensen M. P. Moore M. R. Bockow T. B. Ehde D. M. Engel J. M. (2011). Psychosocial factors and adjustment to chronic pain in persons with physical disabilities: a systematic review. *Arch. Phys. Med. Rehabil.* 92 146–160. 10.1016/j.apmr.2010.09.021 21187217 PMC3028590

[B27] Jöud A. Björk J. Gerdle B. Grimby-Ekman A. Larsson B. (2017). The association between pain characteristics, pain catastrophizing and health care use - Baseline results from the SWEPAIN cohort. *Scand. J. Pain* 16 122–128. 10.1016/j.sjpain.2017.04.071 28850387

[B28] Kurtz S. Ong K. Lau E. Mowat F. Halpern M. (2007). Projections of primary and revision hip and knee arthroplasty in the United States from 2005 to 2030. *J. Bone Joint Surg. Am*. 89 780–785. 10.2106/JBJS.F.00222 17403800

[B29] Landmark T. Dale O. Romundstad P. Woodhouse A. Kaasa S. Borchgrevink P. C. (2018). Development and course of chronic pain over 4 years in the general population: The HUNT pain study. *Eur. J. Pain* 22 1606–1616. 10.1002/ejp.1243 29754398

[B30] Lazaridou A. Kim J. Cahalan C. M. Loggia M. L. Franceschelli O. Berna C. (2017). Effects of Cognitive-Behavioral Therapy (CBT) on Brain Connectivity Supporting Catastrophizing in Fibromyalgia. *Clin. J. Pain* 33 215–221. 10.1097/AJP.0000000000000422 27518491 PMC5296218

[B31] Lazaridou A. Martel M. O. Cornelius M. Franceschelli O. Campbell C. Smith M. (2019). The Association Between Daily Physical Activity and Pain Among Patients with Knee Osteoarthritis: The Moderating Role of Pain Catastrophizing. *Pain Med.* 20 916–924. 10.1093/pm/pny129 30016486 PMC6497093

[B32] Leeuw M. Goossens M. E. Linton S. J. Crombez G. Boersma K. Vlaeyen J. W. (2007). The fear-avoidance model of musculoskeletal pain: current state of scientific evidence. *J. Behav. Med.* 30 77–94. 10.1007/s10865-006-9085-0 17180640

[B33] Leung L. (2012). Pain catastrophizing: an updated review. *Ind. J. Psychol. Med.* 34 204–217. 10.4103/0253-7176.106012 23441031 PMC3573569

[B34] Li R. Sun J. Hu H. Zhang Q. Sun R. Zhou S. (2020). Research Trends of Acupuncture Therapy on Knee Osteoarthritis from 2010 to 2019: A Bibliometric Analysis. *J. Pain Res.* 13 1901–1913. 10.2147/JPR.S258739 32801848 PMC7394582

[B35] Majumder M. Ahmed S. Shazzad N. Hasan A. Haq S. A. Rasker J. J. (2020). Translation, cross-cultural adaptation and validation of the Pain Catastrophizing Scale (PCS) into Bengali in patients with chronic non-malignant musculoskeletal pain. *Int. J. Rheum. Dis.* 23 1481–1487. 10.1111/1756-185X.13954 32862495 PMC7754436

[B36] Martinez-Calderon J. Jensen M. P. Morales-Asencio J. M. Luque-Suarez A. (2019). Pain Catastrophizing and Function In Individuals With Chronic Musculoskeletal Pain: A Systematic Review and Meta-Analysis. *Clin. J. Pain* 35 279–293. 10.1097/AJP.0000000000000676 30664551

[B37] Martín-Martín A. Orduna-Malea E. Thelwall M. Delgado López-Cózar E. (2018). Google scholar, web of science, and scopus: a systematic comparison of citations in 252 subject categories. *J. Informetr.* 12 1160–1177. 10.1016/j.joi.2018.09.002

[B38] Millere A. Kalnberza-Ribule Z. Mezals M. Nulle A. Millere I. Deklava L. (2020). Disability, pain catastrophizing and stress coping of patients with low back pain in rehabilitation practice in Latvia. *J. Back Musculoskelet. Rehabil.* 33 323–328. 10.3233/BMR-170945 31524136

[B39] Penhoat M. Saraux A. Le Goff B. Augereau P. Maugars Y. Berthelot J. M. (2014). High pain catastrophizing scores in one-fourth of patients on biotherapy for spondylarthritis or rheumatoid arthritis. *Joint Bone Spine* 81 235–239. 10.1016/j.jbspin.2013.10.004 24321439

[B40] Pielech M. Ryan M. Logan D. Kaczynski K. White M. T. Simons L. E. (2014). Pain catastrophizing in children with chronic pain and their parents: proposed clinical reference points and reexamination of the Pain Catastrophizing Scale measure. *Pain* 155 2360–2367. 10.1016/j.pain.2014.08.035 25180013 PMC4253605

[B41] Quartana P. J. Campbell C. M. Edwards R. R. (2009). Pain catastrophizing: a critical review. *Expert Rev. Neurother.* 9 745–758. 10.1586/ern.09.34 19402782 PMC2696024

[B42] Roelofs J. Peters M. L. McCracken L. Vlaeyen J. (2003). The pain vigilance and awareness questionnaire (PVAQ): further psychometric evaluation in fibromyalgia and other chronic pain syndromes. *Pain* 101 299–306. 10.1016/S0304-3959(02)00338-X 12583873

[B43] Romeo A. Tesio V. Ghiggia A. Di Tella M. Geminiani G. C. Farina B. (2021). Traumatic experiences and somatoform dissociation in women with fibromyalgia. *Psychol. Trauma* [Preprint]. 10.1037/tra0000907 33646804

[B44] Salt E. Wiggins A. T. Hooker Q. Crofford L. Rayens M. K. Segerstrom S. (2018). The Effects of Pain Severity, Pain Catastrophizing, Depression, and Exercise on Perceived Disability in Acute Low Back Pain Patients. *Res. Theory Nurs. Pract.* 32 436–448. 10.1891/1541-6577.32.4.436 30567914

[B45] Scarone P. Smeets A. van Kuijk S. van Santbrink H. Peters M. Koetsier E. (2020). A randomized controlled TRIal of cognitive BEhavioral therapy for high Catastrophizing in patients undergoing lumbar fusion surgery: the TRIBECA study. *BMC Musculoskel. Dis.* 21:810. 10.1186/s12891-020-03826-w 33276768 PMC7718692

[B46] Sewell M. Churilov L. Mooney S. Ma T. Maher P. Grover S. R. (2018). Chronic pelvic pain - pain catastrophizing, pelvic pain and quality of life. *Scand. J. Pain* 18 441–448. 10.1515/sjpain-2017-0181 29794266

[B47] Sullivan M. J. Thorn B. Haythornthwaite J. A. Keefe F. Martin M. Bradley L. A. (2001). Theoretical perspectives on the relation between catastrophizing and pain. *Clin. J. Pain* 17 52–64. 10.1097/00002508-200103000-00008 11289089

[B48] Sullivan M. Bishop S. R. Pivik J. (1995). The pain catastrophizing scale: development and validation. *Psychol. Assess.* 7 524–532. 10.1037//1040-3590.7.4.524

[B49] Sullivan M. Lynch M. E. Clark A. J. (2005). Dimensions of catastrophic thinking associated with pain experience and disability in patients with neuropathic pain conditions. *Pain* 113 310–315. 10.1016/j.pain.2004.11.003 15661438

[B50] Süren M. Okan I. Gökbakan A. M. Kaya Z. Erkorkmaz U. Arici S. (2014). Factors associated with the pain catastrophizing scale and validation in a sample of the Turkish population. *Turk. J. Med. Sci.* 44 104–108. 10.3906/sag-1206-67 25558568

[B51] Tagliaferri S. D. Miller C. T. Owen P. J. Mitchell U. H. Brisby H. Fitzgibbon B. (2020). Domains of Chronic Low Back Pain and Assessing Treatment Effectiveness: A Clinical Perspective. *Pain Pract.* 20 211–225. 10.1111/papr.12846 31610090

[B52] Taub C. J. Sturgeon J. A. Johnson K. A. Mackey S. C. Darnall B. D. (2017). Effects of a Pain Catastrophizing Induction on Sensory Testing in Women with Chronic Low Back Pain: A Pilot Study. *Pain Res. Manage.* 2017:7892494. 10.1155/2017/7892494 28348505 PMC5350337

[B53] Thomtén J. Boersma K. Flink I. Tillfors M. (2016). Social Anxiety, Pain Catastrophizing and Return-To-Work Self-Efficacy in chronic pain: a cross-sectional study. *Scand. J. Pain* 11 98–103. 10.1016/j.sjpain.2015.10.005 28850478

[B54] Tran B. X. Pham T. V. Ha G. H. Ngo A. T. Nguyen L. H. Vu T. (2018). A Bibliometric Analysis of the Global Research Trend in Child Maltreatment. *J. Environ. Res. Public Health* 15:1456. 10.3390/ijerph15071456 29996540 PMC6069266

[B55] Varallo G. Giusti E. M. Scarpina F. Cattivelli R. Capodaglio P. Castelnuovo G. (2020). The Association of Kinesiophobia and Pain Catastrophizing with Pain-Related Disability and Pain Intensity in Obesity and Chronic Lower-Back Pain. *Brain Sci.* 11:11. 10.3390/brainsci11010011 33374178 PMC7823580

[B56] Varallo G. Scarpina F. Giusti E. M. Cattivelli R. Guerrini Usubini A. Capodaglio P. (2021a). Does Kinesiophobia Mediate the Relationship between Pain Intensity and Disability in Individuals with Chronic Low-Back Pain and Obesity? *Brain Sci.* 11:684. 10.3390/brainsci11060684 34067433 PMC8224628

[B57] Varallo G. Scarpina F. Giusti E. M. Suso-Ribera C. Cattivelli R. Guerrini Usubini A. (2021b). The Role of Pain Catastrophizing and Pain Acceptance in Performance-Based and Self-Reported Physical Functioning in Individuals with Fibromyalgia and Obesity. *J. Pers. Med.* 11:810. 10.3390/jpm11080810 34442454 PMC8401554

[B58] Walid M. S. Donahue S. N. Darmohray D. M. Hyer L. A. Jr. Robinson J. S. Jr. (2008). The fifth vital sign–what does it mean? *Pain Pract.* 8 417–422. 18662363 10.1111/j.1533-2500.2008.00222.x

[B59] Wood T. J. Gazendam A. M. Kabali C. B. Hamilton Arthroplasty, and Group. (2021). Postoperative Outcomes Following Total Hip and Knee Arthroplasty in Patients with Pain Catastrophizing, Anxiety, or Depression. *J. Arthroplasty* 36 1908–1914. 10.1016/j.arth.2021.02.018 33648844

[B60] Woolf C. J. (2011). Central sensitization: implications for the diagnosis and treatment of pain. *Pain* 152(Suppl. 3) S2–S15. 10.1016/j.pain.2010.09.030 20961685 PMC3268359

[B61] Yap J. C. Lau J. Chen P. P. Gin T. Wong T. Chan I. (2008). Validation of the Chinese Pain Catastrophizing Scale (HK-PCS) in patients with chronic pain. *Pain Med.* 9 186–195. 10.1111/j.1526-4637.2007.00307.x 18298701

[B62] Zhu X. Hu J. Deng S. Tan Y. Qiu C. Zhang M. (2021). Bibliometric and Visual Analysis of Research on the Links Between the Gut Microbiota and Depression From 1999 to 2019. *Front. Psychiatry* 11:587670. 10.3389/fpsyt.2020.587670 33488420 PMC7819979

